# A Systematic Review on Caries Status of Older Adults

**DOI:** 10.3390/ijerph182010662

**Published:** 2021-10-12

**Authors:** Alice Kit Ying Chan, Manisha Tamrakar, Chloe Meng Jiang, Edward Chin Man Lo, Katherine Chiu Man Leung, Chun Hung Chu

**Affiliations:** Faculty of Dentistry, The University of Hong Kong, Hong Kong, China; dralice@hku.hk (A.K.Y.C.); manee626@hku.hk (M.T.); cmjiang@hku.hk (C.M.J.); hrdplcm@hku.hk (E.C.M.L.); kcmleung@hku.hk (K.C.M.L.)

**Keywords:** older adult, elderly, oral health, prevention, silver diamine fluoride, caries

## Abstract

The aim of this systematic review was to provide an update on caries prevalence in older adults aged 60 years or above around the globe. Two independent reviewers performed a systematic literature search of English publications from January 2016 to December 2020 using Pubmed, Scopus, Embase/Ovid and Web of Science. The MeSH terms used were “dental caries”, “root caries”, “DMF index”, “aged” and “aged 80 and over”. Further searches in Google Scholar retrieved eight additional publications. The epidemiological surveys reporting the prevalence of dental caries or root caries or caries experience using DMFT (decayed, missing and filled teeth) and DFR (decayed and filled root) in older adults aged 60 years or above were included. Quality of the publications was assessed using the JBI Critical Appraisal Checklist for Studies Reporting Prevalence Data. Among the 5271 identified publications, 39 articles of moderate or good quality were included. Twenty studies were conducted in Asia (China, India, Vietnam, Singapore and Turkey), ten in Europe (Ireland, Norway, Finland, Germany, Portugal, Poland, Romania and Kosovo), three in North America (USA and Mexico), one in South America (Brazil), two in Oceania (Australia) and three in Africa (Malawi, Egypt and South Africa). The prevalence of dental caries ranged from 25% (Australia) to 99% (South Africa), while the prevalence of root caries ranged from 8% (Finland) to 74% (Brazil) in community dwellers. The situation was even worse in institutionalised older adults of which the mean DMFT score varied from 6.9 (Malawi) to 29.7 (South Africa). Based on the included studies published in the last 5 years, caries is still prevalent in older adults worldwide and their prevalence varies across countries.

## 1. Introduction

Dental caries is one of the major oral diseases which cause pain and infection [1] and can impede work productivity in adults [2]. The consequence of severe dental caries is tooth loss which impacts negatively on individual’s aesthetics, function, self-esteem and quality of life [3]. A systematic review on the global burden of untreated caries between 1990 and 2010 reported a high caries prevalence worldwide, affecting 2.4 billion people [4]. This induced a major biological, financial and social burden on individuals, health systems and societies. It also highlighted the trend of caries that shifted from children to adults with the third peak at around the age of 70, due to the appearance of root caries [4].

In 2019, the United Nations estimated that the aging population will be doubled by 2039, with one in six persons at the age of 65 or more [5]. With increasing life expectancy, people retain their teeth for longer [6]. It is expected to see a further increase in untreated caries in this growing population. Ageing, multimorbidity and polypharmacy may increase caries risk in the older adults [7]. Their underlying medical conditions, functional disability and cognitive impairment make dental treatment highly challenging, and unavoidably increase the burden in our health care system [7].

Caries is a preventable disease and various preventive measures are available [8]. In the planning of oral health promotion and prevention programme, an understanding of the current global caries burden is vital. The World Health Organization (WHO) recommends that clinical oral health surveys should be conducted every five to six years within the same community to provide effective surveillance on disease patterns and trends [9]. The decision makers and health authorities can hence formulate policies and develop programmes to prevent and control the disease and conduct evaluations regularly. However, the most recent systematic review of caries status in global population was conducted more than a decade ago, and so far there have been none conducted in older adults [4]. Updated information on caries prevention and control in older adults to facilitate policy planning for the coming decade is needed. The aim of this systematic review is to explore updated information of caries status of older adults globally.

## 2. Materials and Methods

This systemic review was conducted and reported according to the Preferred Reporting Items for Systematic Review and Meta-analysis (PRISMA) guidelines. The protocol was registered in the PROSPERO database (registration number: CRD42021270000).

### 2.1. Search Strategy

Five electronic databases (Pubmed, Scopus, Embase/Ovid, Web of Science and Google Scholar) were searched for articles published from January 2016 to December 2020. The search strategy used in Pubmed was ((prevalence) AND ((((((caries) OR (dental caries)) OR (tooth decay)) OR (root caries)) OR (DMF)))) AND (((((elderly) OR (older)) OR (aged)) OR (aging)) OR (senior)). The combination of keywords such as “prevalence”, “dental caries”, “root caries”, “DMF index”, “aged” and “aged 80 and over” were used for other databases. Search strategy was presented in Supplementary file S1. Duplicates and articles published in languages other than English were excluded. Additional searches were conducted in Google Scholar. The last search date was 17 March 2021. A manual search was performed on the reference list of the included articles.

### 2.2. Study Selection

Two reviewers (AKYC and MT) performed the search independently based on the following selection criteria:

(a) Study design: Epidemiological surveys investigating the prevalence of dental caries or root caries; and baseline findings from longitudinal studies. For multiple publications reporting findings from the same cohort, only the one with the largest sample size was included. Other types of studies such as case reports, literature reviews, letter, commentaries, case–control studies or studies analysing secondary data were excluded:

(b) Participants: older adults aged 60 or above;

(c) Outcomes: dental or root caries prevalence or experience using DMFT (decayed, missing and filled teeth) and DFR (decayed and filled root).

**Figure 1:**
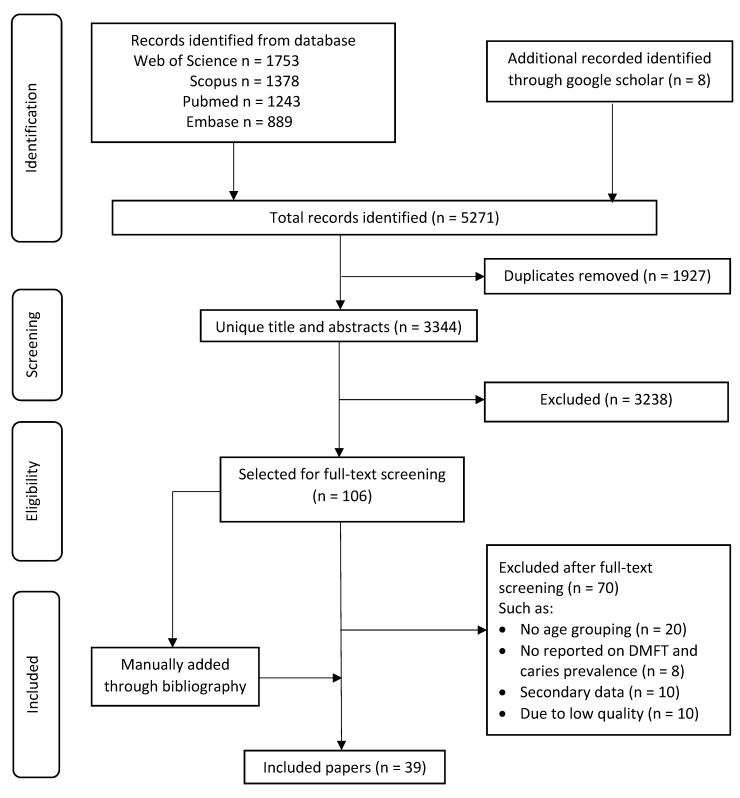
Flowchart for the literature search.

The titles and abstracts of all identified articles were screened. After removal of duplicates, full text of the eligible studies or those that could not be decided by screening the titles and abstracts were retrieved for further assessment. A third reviewer (CMJ) was consulted to make a final decision if there was disagreement between the two reviewers. A flowchart for the literature search is shown in Figure 1

### 2.3. Data Extraction and Quality Assessment

Data which include author, year of publication, country of the study, sampling method, sample size, dental or root caries prevalence, and dental or root caries experience in terms of DMFT or DMFS or DFR or RDFS, mean number of decayed teeth (DT) and diagnostic criteria were extracted on a pre-defined spreadsheet. Data were recorded separately for dental caries and root caries and grouped according to the continents.

Two reviewers independently assessed the quality of the included studies using the JBI Critical Appraisal Checklist for Studies Reporting Prevalence Data (Supplementary file S2) [10]. It consisted of nine questions with a score between 0 and 9. The nine questions were:

1. Sample frame: Was the sample frame appropriate to address the target population?
2. Sampling method: Were study participants sampled in an appropriate way?
3. Sample size: Was the sample size adequate?
4. Study setting: Were the study subjects and the setting described in detail?
5. Sample coverage: Were the data analysed with sufficient coverage of the sample?
6. Measure method: Were valid methods used for the identification of the condition?
7. Outcome reliability: Were the measures for participants set out in a standard and reliable way?
8. Statstical analysis: Was there appropriate statistical analysis?
9. Response rate: Was the response rate adequate, and if not, was the low response rate managed appropriately?

Studies that used a representative sample of the targeted population, used random sampling method, had an adequate sample size estimation, had a good response rate more than 80% or established comparability between respondents and non-respondents, used well-established diagnosis criteria, had independent blind assessment with a good reliability between examiners at kappa value more than 0.6 and adopted appropriate statistical methods were rated as a full score of 9. The quality of the studies was categorised as poor (0–3), moderate (4–6) and good (7–9). Studies scoring 3 or below were excluded.

Preferred Reporting Items for Systematic Reviews and Meta-Analyses (PRISMA) was used as a basis for reporting in this systematic review.

## 3. Results

The two independent reviewers performed data extraction with 97% agreement on the paper they independently selected. The search yielded 5271 publications (1243 from Pubmed, 1378 from Scopus, 889 from Embase and 1753 from Web of Science) in total. Further searches were conducted in Google Scholar for an additional 8 publications. After screening the title and abstract, 1927 publications were duplicates and 3237 of them did not meet the inclusion criteria. Full-text assessment was performed on the remaining 107 publications, and finally 39 studies were included for data extraction.

The included studies reported caries prevalence or experience of older adults at the age of 60 or above in 20 countries from six continents (Table 1). Most of them were conducted in Asia (*n* = 20, China [11,12,13,14,15,16], India [17,18,19,20,21,22,23,24], Singapore [25], Turkey [26,27,28,29,30,31,32,33,34,35,36,37,38] and Vietnam [29,30]) and Europe (*n* = 10, Finland [31], Germany [32,33], Ireland [34], Kosovo [35], Norway [36], Poland [37,38], Portugal [39] and Romania [40]). There were three studies conducted in North America (Mexico [41,42] and USA [43]), one in South America (Brazil [44]), two in Oceania (Australia [45,46]) and three in Africa (Egypt [47], Malawi [48] and South Africa [49]). A summary of the included studies was presented in Table 2.

Most of the studies (28/39) were conducted on community dwellers in community setting. The rest were carried out in residential homes or day centres (10 studies [13,14,16,17,19,20,30,39,41,42]). One study was hospital-based [25]. One study was conducted on home bound older adults with functional disability [31].

The untreated caries and root caries prevalence and experience reported from the included studies were summarised in Table 3 and Table 4, respectively. Among the 39 included studies, 33 of them reported untreated caries status, whereas 11 of them reported root caries status. For caries diagnostic criteria, majority of the included studies (33/39) used the WHO criteria, one study each used ICDAS [16], Banting [17] and Barmes [33] criteria and a 5-grade scale [36], while two studies [40,43] did not specify how caries was diagnosed.

Among the included studies, twenty-six of them reported the prevalence of untreated caries and/or root caries. Untreated caries varied among continents, with the highest prevalence found in Asia and Africa; the majority of the studies reported caries prevalence of 50% or more in their older adult population. The lowest prevalence of untreated caries was reported in Australia. Caries prevalence also varied between community dwellers and institutionalised older adults. The prevalence of untreated caries ranged from 25% (Australia) to 99% (South Africa) in community dwellers and from 47% (India) to 99% (Vietnam) in institutionalised older adults. The global median of mean prevalence of caries was 49%. Eleven studies reported the prevalence of untreated root caries with half of them conducted in residential home and/or day care centres. The prevalence of untreated root caries ranged from 8% (Finland) to 74% (Brazil) in community dwellers, and from 30% (Hong Kong) to 96% (Vietnam) in institutionalised older adults. The median of mean prevalence of root caries was 46%. The median of mean prevalence of untreated and root caries among continents was presented in Table 1.Twenty-seven studies reported caries experience at tooth level using DMFT/DFR while two studies from Australia reported that at surface level using DMFS/RDFS. The mean DMFT varied from 6.9 in Malawi to 29.7 in South Africa. The global median of mean DMFT score was 21.9. The mean DT varied from 0.3 in South Africa to 6.4 in Vietnam with a median of 1.65. Nine studies did not report caries experience but prevalence of caries only. Twelve studies also investigated the socioeconomic and behavioural risk factors of caries in older adults (Table 5). Household income (6/12) and education level (4/12) were mostly reported as socioeconomic risk factors while frequency of tooth brushing (6/12)], dietary habits (4/12) and frequency of dental visits (3/12) were found as behavioural risk factors of caries in older adults.

Among the 39 included studies, 17 of them were rated as good quality whereas 22 were rated as moderate quality under the JBI Critical Appraisal Checklist for Studies Reporting Prevalence Data. The quality assessment of the included studies was presented in Table 6.

## 4. Discussion

Oral disease was the fourth most expensive disease to treat [50]. In the last decade, untreated caries was prevalent worldwide, affecting 2.4 billion people with the third peak at the age of 70 [4]. The situation remains the same after a decade, as reflected from the results of our study. Our result showed that untreated caries was still widespread globally in older adults. The majority of the included studies reported a prevalence of untreated caries of 50% or more. It varied among continents with the highest prevalence in Asia and Africa and the lowest in Australia. The median of the mean number of teeth with untreated caries was 1.55 per older adult around the globe.

This study included published national-based cross-sectional surveys; however, those which have not been published were excluded. There were two national oral health surveys published in English and conducted in the past five years, one in China and one in Australia. This study selected publications in English, and we could potentially neglect including useful non-English publications such as government reports and national surveys published in other languages. However, we found a high risk of errors in translation, particularly for the many free online tools. Selecting only English publications in this study also allowed us to standardise the search protocol for the two independent researchers in the literature search. Some surveys may not provide results in the database search and in the Google scholar search. This can be a limitation of this study, but it is difficult to generate a list of databases with different languages.

The reported prevalence of untreated caries in older adults in China was similar to that in 2005 (98% vs. 98.4%) [51]. Although Australia has the lowest caries prevalence worldwide, the prevalence of untreated caries in older adults reported in this study was found higher than that in 2004–2006 (25% vs. 22%) [52]. There was not much improvement in caries status in the older adult population during the past decade, even though the concern about the global burden of dental caries has been repeatedly emphasized.

The 2003 WHO oral health report emphasized the need to relieve the burden of caries in children and proposed to implement a number of strategies for the promotion of oral health through health programmes and educational activities at schools [53]. With immense efforts from workers of healthcare and other sectors, caries prevalence in children is reducing. In contrast, the burden of caries has been shifted to adults in the last decade [4]. In Europe, the trend in caries status in the older adult population is different from that of the younger generation. A systematic review found that Europe showed the lowest prevalence of early childhood caries than that of other continents [54]. However, in our study, older adults in Europe did not have the lowest caries prevalence but was higher than that of North America and Oceania, and the mean DMFT score (23.4) was the highest amongst others. Our findings were in line with another systematic review which reported a decline in caries in European adults in the past two decades, but it was just to a minor extent in the senior citizens [55]. Caries in older adults is still a major public health issue, even in developed countries. There is a need to extend oral health promotion and education activities across the entire lifespan.

The mean DMFT score reported in the included studies comprised a large portion of mean number of missing teeth (MT) but a low portion of mean number of filled teeth (FT). This indicates that treatment of dental diseases is mainly dealt with by extraction of unsalvageable teeth rather than fillings to manage pain and oral infection, and to restore carious teeth. Such observation may imply that the dentition may subject to recurrent caries attacks and eventually becomes unrestorable despite repeated fillings. In addition, due to high treatment cost and limited dental access, extraction could be a more preferable option. A shift in DMFT components from missing teeth (MT) to filled teeth (FT) has been noticed in older adults living in European countries in the past decade [55]. This indicates that treatment approach for caries is shifted from extraction to restorative intervention due to improved dental health services. However, extraction is still common in most developing countries nowadays as some of our included studies showed an extremely high MT score [22,23,28,29,35,48,49]. Resources are still limited in these countries and people may have difficulties in gaining access to dental service or affording the treatment cost [48,49]. At a result, they tend to seek dental treatment when they experience severe pain and extraction may be the only option as caries have progressed to an advanced stage [35].

It is interesting to note that the mean DMFT score was comparatively low as revealed in the national-based oral survey conducted in Malawi [48]. It was the lowest among our included studies and lower than those reported in previous study conducted in another sub-Saharan Africa country [56]. Malawi is among the least developed countries in the world with most of the population living in rural areas. Although the authors did not provide any explanation to the low mean DMFT score, they reported that three-fourths of the older adults brushed their teeth twice daily, which was the highest among the included studies. The importance of good oral hygiene habits and high dental awareness cannot be overemphasized for good oral health.

Socioeconomic factors play an important role in caries development [57]. Some of these factors are behavioural-dependent and are modifiable through changing lifestyle and behavioural habits [57]. Understanding the risk factors of caries in older adults is essential for planning oral health education and prevention programmes. However, there were only a few included studies further investigated on the associated risk factors. Low education level and income level were reported to be the socioeconomic risk factors associated with caries development in older adults. The behavioural habits such as frequency of tooth brushing, dietary habit and dental attendance also affected the chance of having caries. Among the included studies, eight studies investigated the association between plaque control and caries. Six studies revealed that reduced toothbrushing frequency to once per day [11,17,36], twice per week [44] or no toothbrushing at all [39,47] increased the risk. A systematic review found similar results that people who brushed their teeth infrequently were at higher risk for incidence of new caries than those who brushed frequently [58]. Three of the included studies found that older adults who did not pay an annual dental visit had increased risk of having caries [12,36,46]. Although four studies claimed that dietary habits affected caries risk, they were looking at different types of diet that we cannot easily draw a conclusion from [12,17,36,47]. Behavioural and socioeconomic risk factors should be included in the investigation in epidemiological studies on caries status of older adults in the future.

There are three peaks of caries development throughout the lifespan and the third one appeared at 70 years old, with the appearance of root caries [4]. However, root caries status in older adults has not been widely studied. Less than one-third of our included studies reported root caries status in older adults and was limited to prevalence only. More studies are needed to provide information of root caries status in older adults with details such as the number of sites and affected surfaces so that effective prevention and treatment strategies can be planned in preparation of the increasing number of untreated root caries in the foreseeable future.

### 4.1. Strengths and Limitations in This Study

Our study has several strengths. We used four databases (Pubmed, Scopus, Embase/Ovid and Web of Science) and Google Scholar to identify as many relevant publications as we can according to the keywords used. All the searching, quality assessment and data extraction were performed by two independent assessors with 97% agreement on the paper they independently selected. In order to include papers with better quality, the JBI Critical Appraisal Checklist for Studies Reporting Prevalence Data, the newest quality assessment tool for purely descriptive cross-sectional study [59], was used for assessment and those categorised as low quality were excluded. The aim of this study was to update information on caries status in older adults, hence only studies published within the previous five years, 2016–2020, were included. We included those reporting the prevalence of caries and/or experience using DMFT/DMFS/DFR/RDFS index as these are the most commonly used parameters in reporting caries status. More importantly, it is the first systematic review on global caries status in older adults and hence can provide a baseline data for policy makers and researchers on health policy planning.

We came across certain limitations in this study. Although we did a comprehensive search of a few databases, studies about caries status of older adults were scarce especially in South America. Because of the scarcity of the studies, we did not impose sample size requirement for inclusion in order to cover more studies for data extraction. However, bias may exist when the results from those with small sample size was reported. To increase the number of studies included in this review, we included studies used different indices to define dental caries such as DMFT and ICDAS. This may influence the diagnosis of the pathology and its prevalence. Furthermore, the caries prevalence reported could be affected by the different setting and the different target population of the studies. Moreover, meta-analysis could not be conducted due to the heterogeneity among the studies. Medians rather than means of prevalence and DMFT score were reported. Further, since the associated risk factors were assessed differently among studies and different categories of risk factors were used, we could only provide a qualitative description rather than a quantitative analysis.

### 4.2. Implications

This study highlights to health professionals and policymakers that the prevalence of dental caries is still high among older adults in many countries, particularly in Asia and Africa, with a caries prevalence of 50% or more. It is expected that when they further advance in age, the caries risk would increase due to deterioration in health and comorbidities at the last years of life. National and global oral health policy should emphasize oral health promotion and prevention throughout the entire lifespan.

Caries is a preventable disease. There are different preventive measures available. Fluoride therapy has long been proved to be effective in caries prevention [60]. Silver diamine fluoride (SDF) has been proven to be effective in caries arrest and prevention in children [61]. A recent systematic review showed that SDF was effective on root caries prevention in older adults [62]. High dose fluoride toothpaste is another fluoride agent recommended for caries management in older adults [63]. More well-designed clinical trials should be conducted to provide information on their clinical uses in older adults. A recent study showed root caries was associated with candidal infection; this may complicate caries management [64]. This implied further investigation in inhibiting the growth of candidal biofilm may be beneficial in caries prevention in the future [65].

Our study also points out that older adults with low education level and insufficient oral health knowledge in terms of oral hygiene practice and dietary habit had increased risk of caries development. Population-based oral health promotion programmes should be performed regularly to educate older adults and their family members or care givers in caries prevention. Outreach dental services can be provided where health professionals visit day care centres or residential homes regularly to extend the oral health promotion activities to cover those with functional or physical disability. Oral health promotion and prevention programmes in older adults can be integrated with other health sectors to improve both oral and general health.

Social determinants of health have been well documented for a long time but implementation of policy to address them is still lacking [66]. It is shown in our study that current dental care is still intervention-oriented which can be unaffordable by patients with underprivileged background [67]. Health professionals and governments should work together in shifting dental care to a prevention-oriented approach with emphasis on regular dental check-ups, oral health education promotion and cost-effective method on caries prevention and control [67]. With this widespread policy change, we hope we can tackle this long-standing oral health inequality in the coming decade.

Our study had some implications for researchers. Firstly, there were lack of periodic and properly conducted oral health surveys reporting the caries status in older adult population worldwide when compared with other age groups. Most of them were regional-based rather than national-based. More regular national-based oral health surveys are needed to monitor the caries trend and pattern of caries in this age group worldwide so as to understand the global need for better policy planning. Secondly, there were insufficient data about the caries status in institutionalised older adults who have higher caries risk due to their dependency, multimorbidity and polypharmacy when compared with independent community dwellers [7]. With the increasing aging population and life expectancy, increasing number of institutionalised older adults is expected. More data to understand their need is needed to tailor a suitable oral health programme for them. Thirdly, caries status was reported mainly in two categories, caries prevalence and caries experience. However, different parameters were used such as DMFT, DMFS, DFR, RDFS and percentage of caries among studies. This makes direct comparison among different populations difficult. Future epidemiological surveys should consider using common diagnostic criteria and measuring parameters so that comparisons with previous studies are possible. Fourthly, definition of caries severity in older adults should be developed. At present, WHO categorises DMFT > 4.5 and DMFT > 13.9 as a high level of caries experience in children (below 12) and adults (35–44), respectively; however, no definition has been established for application to the older adult group [9]. With a clear definition, policymakers and general population would have an easier understanding of caries severity and health professionals would have a clear goal to achieve. Lastly, as older adults are expected to have their third peak of caries attack at 70 [4], likely happens as root caries, oral health surveys should also describe their root caries status as well as the associated risk factors so that better insight on root caries prevalence and severity in older adults can be gained.

## 5. Conclusions

Based on the included studies published in the past 5 years (2016–2020), the prevalence of caries in older adults was still high in most countries around the globe. The health policy makers should have better planning to relieve the increasing global burden of caries from the surging older adult population in the coming decade.

## Figures and Tables

**Table 1:** The median of mean prevalence of caries among continents.

Location	Median of Untreated Caries Prevalence	Median of Root Caries Prevalence
Global (20 countries, 39 studies)	49%	46%
Asia (5 countries, 20 studies)	66%	46%
Europe (8 countries, 10 studies)	46%	35%
North America (2 countries, 3 studies)	25%	95.3%
South America (1 country, 1 study)	-	74%
Oceania (1 country, 2 studies)	25%	18%
Africa (3 countries, 3 studies)	49%	-

**Table 2:** Summary of the included studies: Study origin, sampling, demographics and types of caries reported.

Country [Ref.]	Sampling Method	Setting	Participants	Location	Types of Caries
Asia					
China [11]	Multistage Cluster	Community	65–74 yrs; Female: 65%	Urban: 51%	Coronal
China [12]	Multistage Cluster	Community	65–74 yrs; Female: 65%	Urban: 51%	Root
China [13]	Purposive	Residential home	≥60 yrs; Female: 66%		Coronal
China [14]	Consecutive	Day care centre	≥65 yrs; Female: 76%; Dementia: 54%		Coronal
China [15]	-	Community	65–94 yrs; Female: 68%; Systemic disease: 71%		Coronal
China [16]	Purposive	Community	≥60 yrs; Female: 81%		Root
India [17]	Cluster	Residential home	≥60 yrs; Female: 60%		Root
India [18]	Convenience	Community	≥60 yrs; Female: 30%		Coronal
India [19]	Not reported	Residential home	60–69 yrs; Female: 60%		Coronal and Root
India [20]	Simple Random	Residential home	≥65 yrs; Female: 55%		Coronal
India [21]	Stratified cluster	Community	65–74 yrs	Rural: 98%	Coronal
India [22]	Stratified sampling	Community	≥60 yrs; Female: 51%	Rural: 100%	Coronal
India [23]	Multistage cluster	Community	65–74 yrs; Female: 50%	Rural:100%	Coronal
India [24]	Systematic Random	Community	≥65 yrs	Urban:100%	Coronal
Singapore [25]	Random	Hospital	60–80 yrs; Female: 27%; Psychiatric: 100%		Coronal
Turkey [26]	-	Community	≥65 yrs		Coronal
Turkey [27]	Random	Community	≥60 yrs; Female: 47%; Systematic disease: 90%		Coronal
Turkey [28]	Not Reported	Community	70–80 yrs		Coronal
Vietnam [29]	Multistage Stratified	Community	≥65 yrs; Female: 50%	Rural: 47%	Coronal
Vietnam [30]	-	Residential home	≥60 yrs; Female: 55%; Systematic disease: 51%		Coronal and Root
Europe					
Finland [31]	Random	Community	Age: ≥75; Female: 74; Functional disability: 100%		Coronal and Root
Germany [32]	Census	Community	100 yrs; Female: 84%		Coronal and Root
Germany [33]	Convenience	Community	≥60 yrs; Female: 52%		Coronal
Ireland [34]	Advertisement	Community	≥65 yrs; Female: 56%		Coronal and Root
Kosovo [35]	Not Reported	Community	≥65 yrs		Coronal
Norway [36]	Random	Community	65–79 yrs		Coronal
Poland [37]	Random	Community	65–74 yrs; Female: 52%		Coronal
Poland [38]	Volunteer	Community	≥65 yrs; Female: 64%; Depression: 30%		Coronal
Portugal [39]	Probabilistic	Residential home	≥60 yrs; Female: 70%		Coronal and Root
Romania [40]	Not Reported	Community	65–74 yrs; Female: 55%	Rural: 100%	Coronal
North America					
Mexico [41]	Not Reported	Community	≥60 yrs; Female: 70%		Root
Mexico [42]	Not Reported	Residential home	≥60 yrs; Female: 100%; Systematic disease: 67%	Rural: 42%	Coronal
USA [43]	Not Reported	Community	≥60 yrs; Female: 71%		Coronal
South America					
Brazil [44]	Simple Random	Community	≥60 yrs; Female: 49%; Depression: 11%	Rural: 47%	Root
Oceania					
Australia [45]	Not reported	Community	≥75 yrs		Root
Australia [46]	Not reported	Community	≥75 yrs		Coronal
Africa					
Egypt [47]	Convenience	Community	≥65 yrs		Coronal
Malawi [48]	Multistage	Community	65–74 yrs		Coronal
South Africa [49]	Consecutive	Community	≥65 yrs		Coronal

**Table 3:** Summary of the included studies reported on dental caries.

Caries Prevalence	Sample Size	DMFT ± SD	DT ± SD	Diagnostic Criteria	Country (Ref.)
43–99%	21–4331	9.2–26.7	1–6.4		Asia
99% *	791	-	5.8 ± 4	WHO	Vietnam [30]
98%	4431	13.3 ± 9.3	3.3 ± 4.2	WHO	China [11]
89%	258	14.3 ± 8.7	6.4 ± 5.5	WHO	Vietnam [29]
77%	248	16.4 ± 9	-	WHO	India [18]
76%	352	-	-	WHO	India [22]
56%	165	18.5 ± 13.1	-	WHO	India [21]
49%	192	12 ± 9.7	1.6 ±2.2	WHO	India [23]
47%	195	-	1 ± 1.5	WHO	China [15]
47% *	175	20.3 ± 10.2	2.2 ± 3.6	WHO	India [19]
43%	23	-	-	WHO	India [24]
-	709	19.1 ± 7.3	-	WHO	Turkey [27]
- *	512	15.1 ± 8.2	-	WHO	China [13]
-	392 (65–74)	21.9 ± 5.2	-	WHO	Turkey [26]
-	429 (75+)	23.6 ± 4.7	-		
- *	150	23.9	-	WHO	India [20]
- *	129	22.5 ± 7.9	2.1 ± 3.1	WHO	China [14]
-	99	19.2 ± 9.3	2.3 ± 2.6		
- *	64	26.7 ± 6.4	3.5 ±5.1	WHO	Singapore [25]
-	21	9.2 ± 4.8	1 ± 0.7	WHO	Turkey [28]
21–59%	55–1626	17.8–27.5	0.5–5.3		Europe
59%	196	-	-	-	Romania [40]
50% *	372	25.6 ± 7.3	1.7 ± 2.3	WHO	Portugal [39]
49%	387	17.8	1.1	WHO	Poland [37]
42%	1626 (65–74)	18 ± 9.9	1.1 ± 2	WHO	Kosovo [35]
-	273 (75+)	23.2 ± 9.4	0.5 ± 1.5		
30%	269	-	0.8 ± 1.8	WHO	Finland [31]
-	500	27.5 ± 5	1.5 ± 2.7	WHO	Poland [38]
-	334	23.5 ± 5	-	WHO	Ireland [34]
-	308	22.5	0.8	5-grade scale	Norway [36]
-	61 (migrant)	24.8 ± 3.9	5.3 ± 4.6	Barmes	Germany [33]
-	51(non-migrant)	23.4 ± 4.6	2.1 ± 2.8		
-	55	25.2 ± 3.9	1.2	WHO	Germany [32]
25%	170–512	-	1.9		North America
25%	512	-	-	-	USA [43]
- *	170	-	1.9 ± 3.5	WHO	Mexico [42]
25%	433	-	-		Oceania
25%	433	-	-	WHO	Australia [46]
42–99%	12–683	6.9–30.1	0.2–3.4		Africa
-	243 (65–74)	29.7 ± 5.9	0.3 ± 1.3	WHO	South Africa [49]
99%	74 (75+)	30.1 ± 6.1	0.2 ± 1.6		
49%	683	6.9	1.41	WHO	Malawi [48]
42%	12	11.4 ± 7.6	3.4 ± 7.4	WHO	Egypt [47]

* Study conducted in institutionalised older adults.

**Table 4:** Summary of the included studies reported on root caries.

Root Caries Prevalence	Sample Size	DT ± SD	Diagnostic Criteria	Countries (Ref.)
30–96%	175–4431	0.7–6		Asia
96% *	791	6 ± 4.2	WHO	Vietnam [30]
62%	4431	2.6 ± 3.7	WHO	China [12]
46% *	312	-	Banting	India [17]
40% *	175	-	WHO	India [19]
30% *	353	0.7 ± 1.7	ICDAS	China [16]
8–54%	55–372	0.1–3.4		Europe
54% *	372	3.4 ± 3.6	WHO	Portugal [39]
35%	55	1.1 ± 1.5	WHO	Germany [32]
8%	269	0.1 ± 0.6	WHO	Finland [31]
95%	139			North America
95% *	139	-	WHO	Mexico [41]
74%	390			South America
74%	390	-	WHO	Brazil [44]
18%	433			Oceania
18%	433	-	WHO	Australia [45]

* Study conducted in institutionalised older adults.

**Table 5:** Socioeconomic and behavioural factors related to caries assessed in the studies.

Country of Study [Ref.]	Household Income	Education Level	Living Region	Dietary Habit	Brushing Habit	Dental Visit
Egypt [47]	Y	Y	-	Y	Y	-
Australia [46]	Y	-	Y	-	-	Y
China [12]	-	Y	-	Y	-	Y
Norway [36]	-	-	-	Y	Y	Y
India [17]	Y	-	-	Y	Y	-
Australia [45]	Y	-	Y	-		-
China [11]	Y	-		-	Y	-
Brazil [44]	-	-	Y	-	Y	-
Portugal [39]	-	-	-	-	Y	-
Turkey [28]	-	Y	-	-	-	-
USA [43]	Y		-	-	-	-
India [18]	-	Y	-	-	-	-

**Table 6:** Quality assessment of the studies by continents.

Quality (Score)	Sample Frame	Sampling Method	Sample Size	Study Setting	Sample Coverage	Measure Method	Outcome Reliability	Statistical Analysis	Response Rate	Country [Ref.]
Asia										
Good (8)	Y	Y	Y	Y	-	Y	Y	Y	Y	China [14]
Good (8)	Y	Y	Y	Y	-	Y	Y	Y	Y	China [11]
Good (8)	Y	Y	Y	Y	-	Y	Y	Y	Y	China [12]
Good (8)	Y	Y	Y	Y	-	Y	Y	Y	Y	India [17]
Good (8)	Y	Y	Y	Y	-	Y	Y	Y	Y	Turkey [28]
Good (8)	Y	Y	Y	Y	-	Y	Y	Y	Y	Vietnam [29]
Good (7)	Y	Y	Y	Y	-	Y	-	Y	Y	India [23]
Good (7)	-	Y	Y	Y	-	Y	Y	Y	Y	India [21]
Moderate (6)	-	-	Y	Y	-	Y	Y	Y	Y	China [15]
Moderate (6)	-	Y	Y	Y	-	Y	Y	Y	-	India [19]
Moderate (6)	-	-	Y	Y	-	Y	Y	Y	Y	India [20]
Moderate (6)	Y	Y	Y	Y	-	-	-	Y	Y	India [24]
Moderate (6)	-	-	Y	Y	-	Y	Y	Y	Y	Turkey [26]
Moderate (6)	Y	Y	Y	-	-	Y	-	Y	Y	Vietnam [30]
Moderate (5)	-	-	Y	-	-	Y	Y	Y	Y	China [16]
Moderate (5)	-	-	-	Y	-	Y	Y	Y	Y	China [13]
Moderate (5)	-	-	Y	Y	-	Y	-	Y	Y	India [22]
Moderate (5)	-	Y	-	Y	-	-	Y	Y	Y	Singapore [25]
Moderate (4)	-	-	-	Y	-	Y	Y	Y	-	India [18]
Moderate (4)	-	Y	-	-	-	Y	-	Y	Y	Turkey [27]
Europe										
Good (8)	Y	Y	Y	Y	Y	Y	-	Y	Y	Germany [32]
Good (8)	Y	Y	Y	Y	-	Y	Y	Y	Y	Kosovo [35]
Good (8)	Y	Y	Y	Y	-	Y	Y	Y	Y	Norway [36]
Good (7)	-	Y	Y	Y	-	Y	Y	Y	Y	Finland [31]
Good (7)	-	Y	Y	Y	Y	-	Y	Y	Y	Romania [40]
Moderate (6)	-	-	Y	Y	-	Y	Y	Y	Y	Poland [38]
Moderate (6)	-	-	Y	Y	-	Y	Y	Y	Y	Portugal [39]
Moderate (5)	-	-	-	Y	Y	Y	-	Y	Y	Germany [33]
Moderate (5)	Y	-	-	Y	Y	Y	-	Y	-	Poland [37]
Moderate (4)	-	-	-	Y	-	Y	Y	Y	-	Ireland [34]
North America										
Moderate (6)	Y	Y	Y	-	Y	-	-	Y	Y	USA [43]
Moderate (5)	-	-	-	-	Y	Y	Y	Y	Y	Mexico [41]
Moderate (4)	-	-	-	Y	-	Y	Y	Y	-	Mexico [42]
South America										
Good (8)	Y	Y	Y	Y	-	Y	Y	Y	Y	Brazil [44]
Oceania										
Good (7)	Y	Y	Y	Y	-	Y	Y	-	Y	Australia [45]
Good (7)	Y	Y	Y	Y	-	Y	Y	-	Y	Australia [46]
Africa										
Good (8)	Y	Y	Y	Y	-	Y	Y	Y	Y	Malawi [48]
Moderate (6)	-	Y	Y	Y	-	Y	Y	-	Y	Egypt [47]
Moderate (6)	Y	Y	-	Y	-	Y	Y	Y	-	South Africa [49]

## Data Availability

Not applicable.

## Supplementary Materials

The following are available online at https://www.mdpi.com/article/10.3390/ijerph182010662/s1, Supplementary file S1: Search strategy in all databases, Supplementary file S2: JBI Critical Appraisal Checklist for Studies Reporting Prevalence Data.
