# Supplementary material for: A Systematic Review on Caries Status of Older Adults
Source: Int J Environ Res Public Health. 2021 Oct 12;18(20):10662. doi: 10.3390/ijerph182010662 (PMC8535396; doi:10.3390/ijerph182010662)
Supplement: Supplementary file 1 [file ijerph-18-10662-s001.zip › Supplementary file S1.pdf]

Search strategy in all databases

Pubmed (n=1243)

((prevalence) AND ((((((caries) OR (dental caries)) OR (tooth decay)) OR (root caries)) OR (DMF)))) AND (((((elderly) OR (older)) OR (aged)) OR (aging)) OR (senior)) AND ((2016/1/1:2020/12/31[pdat]) AND (english[Filter]))

Scopus (n= 1378)

((TITLE-ABS-KEY(elderly) OR TITLE-ABS-KEY(senior) OR TITLE-ABS-KEY(agieng) OR TITLE-ABS-KEY(aged) OR TITLE-ABS-KEY(older))) AND (TITLE-ABS-KEY(prevalence)) And ((TITLE-ABS-KEY(caries) OR TITLE-ABS-KEY("dental caries") OR TITLE-ABS-KEY("root caries") OR TITLE-ABS-KEY("tooth decay") OR TITLE-ABS-KEY(DMF))) AND (LIMIT-TO (PUBYEAR,2020) OR LIMIT-TO (PUBYEAR,2019) OR LIMIT-TO (PUBYEAR,2018) OR LIMIT-TO (PUBYEAR,2017) OR LIMIT-TO (PUBYEAR,2016)) AND (LIMIT-TO (LANGUAGE,"English"))

Embase (n=889)

(aged or elderly or ageing or senior or older) and (caries or "dental caries" or "tooth decay" or "root caries" or DMF) and prevalence.af.(english language and yr="2016 - 2020")

WOS (n=1753)

(All=(senior) OR ALL=(aged) OR ALL=(aging) OR ALL=(elderly) OR ALL=(older)) AND (ALL=(caries) OR ALL=("dental caries") OR ALL=("root caries) OR ALL=("tooth decay") OR ALL=("DMF") AND (ALL=prevalence) *AND* **LANGUAGE**: (English)

Indexes=SCI-EXPANDED, SSCI, A&HCI, CPCI-S, CPCI-SSH, ESCI Timespan=2016-2-20
