# Supplementary material for: A Systematic Review on Caries Status of Older Adults
Source: Int J Environ Res Public Health. 2021 Oct 12;18(20):10662. doi: 10.3390/ijerph182010662 (PMC8535396; doi:10.3390/ijerph182010662)
Supplement: Supplementary file 1 [file ijerph-18-10662-s001.zip › Supplementary file S2.pdf]

JBI Critical Appraisal Checklist for Studies Reporting Prevalence Data

The questions are as below. The answer will be 'Yes', 'No', 'Unclear' or 'Not applicable'.

1. Was the sample frame appropriate to address the target population?
2. Were study participants sampled in an appropriate way?
3. Was the sample size adequate?
4. Were the study subjects and the setting described in detail?
5. Were the data analysis conducted with sufficient coverage of the identified sample?
6. Were valid methods used for the identification of the condition?
7. Was the condition measured in a standard, reliable way for all participants?
8. Was there appropriate statistical analysis?
9. Was the response rate adequate, and if not, was the low response rate managed appropriately?

One mark will be given for each question with the answer 'Yes'. The maximum total mark for each study is 9.

The quality of the study will be categorized as low (total score: 0-3), moderate (total score: 4-6) and good (total score: 7-9).

Remarks:

Question 5: If the overall response rate is over 80%, it would be treated as having good response rate.

Question 6: We consider the use of WHO criteria, ICDAS criteria or other validated diagnostic criteria for caries diagnosis as the appropriate diagnostic method.

Question 7: Independent blind assessment with a Kappa value of 0.6 or more on intra-/inter-examiner(s) agreement will be considered as appropriate.

Question 9: Study with a good response rate of 80% or over will be given a 'Yes'. Study without a good response rate but mentioned the reasons of not responding and compare the non-response and response groups will be given a 'Yes' too.
